# The role of imaging in the selection of patients for HFpEF therapy

**DOI:** 10.1093/ehjci/jead137

**Published:** 2023-07-03

**Authors:** Tomasz Baron, Spyridon Gerovasileiou, Frank A Flachskampf

**Affiliations:** Department of Medical Sciences, Cardiology and Clinical Physiology, Uppsala University and Uppsala University Hospital, 751 85 Uppsala, Sweden; Uppsala Clinical Research, 751 85 Uppsala, Sweden; Department of Medical Sciences, Cardiology and Clinical Physiology, Uppsala University and Uppsala University Hospital, 751 85 Uppsala, Sweden; VO Medicin, Lasarettet i Enköping, all 785 81 Uppsala, Sweden; Department of Medical Sciences, Cardiology and Clinical Physiology, Uppsala University and Uppsala University Hospital, 751 85 Uppsala, Sweden

**Keywords:** heart failure with preserved ejection fraction, imaging, echocardiography, cardiovascular magnetic resonance, nuclear imaging, heart failure therapy

## Abstract

Heart failure with preserved ejection fraction (HFpEF) traditionally has been characterized as a form of heart failure without therapeutic options, in particular with a lack of response to the established therapies of heart failure with reduced ejection fraction (HFrEF). However, this is no longer true. Besides physical exercise, risk factor modification, aldosterone blocking agents, and sodium-glucose cotransporter 2 inhibitors, specific therapies are emerging for specific HFpEF etiologies, such as hypertrophic cardiomyopathy or cardiac amyloidosis. This development justifies increased efforts to arrive at specific diagnoses within the umbrella of HFpEF. Cardiac imaging plays by far the largest role in this effort and is discussed in the following review.

## Graphical Abstract

**Graphical Abstract:**
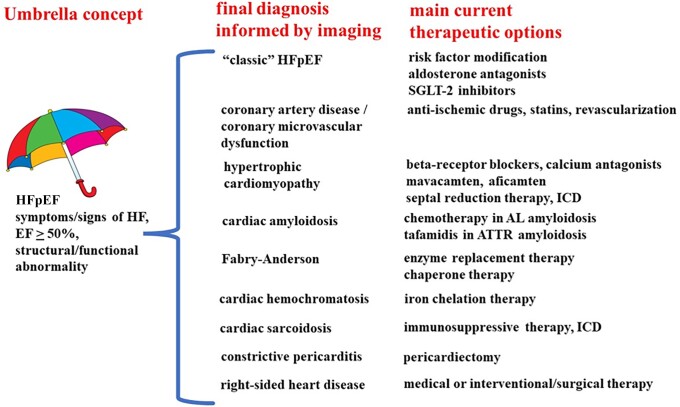

## Introduction

**Figure 1:**
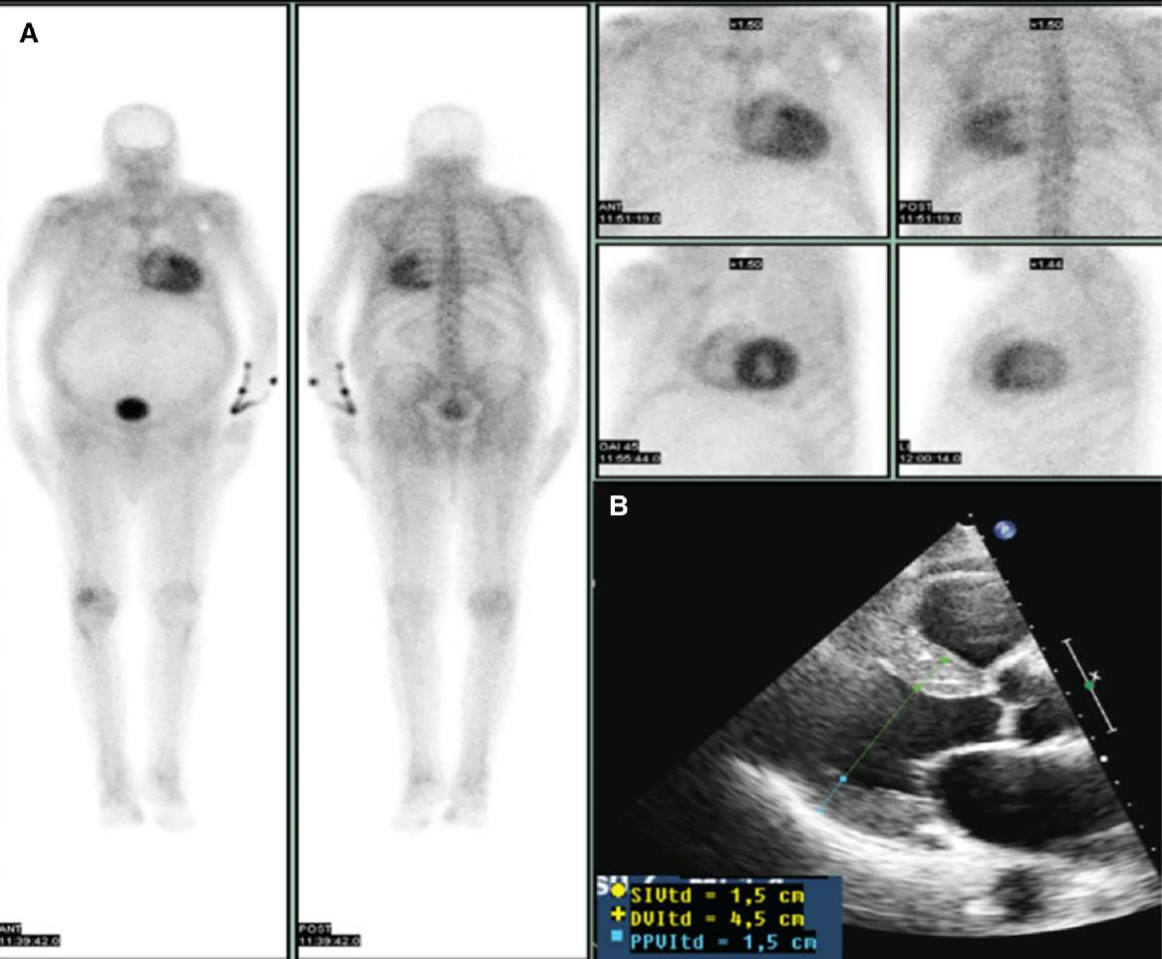
Illustrative example of HFpEF patient in whom evaluation by DPD scintigraphy (left and upper right) revealed ATTR amyloidosis, reproduced with permission from.^1^ Lower right, echocardiographic parasternal long-axis view showing concentric hypertrophy of the left ventricle (septal and posterior wall thickness, both 15 mm). See text for further details.

**Figure 2:**
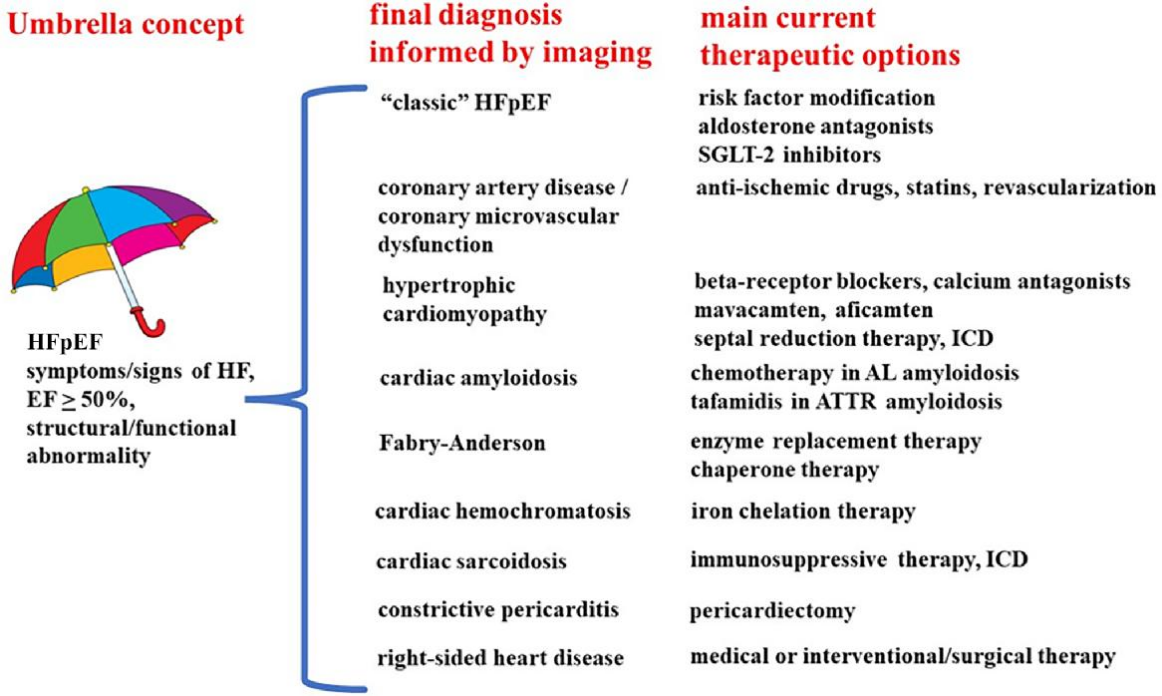
Schematic overview of diseases covered by the “umbrella concept” of HFpEF. Imaging plays a crucial role for their identification and identification of therapeutic option. Rare cardiomyopathies and valvular heart disease have been omitted for the sake of clarity.

In a remarkable study from 2015, González-López et al. sent consecutive patients 60 years or older with newly diagnosed heart failure with preserved ejection fraction (HFpEF) and a septal thickness of at least 12 mm on echocardiography to ^99m^Tc-3,3-diphosphono-1,2-propanodicarboxylic acid (DPD) scintigraphy, and found a rate of 13% of wild-type transthyretin cardiac amyloidosis (*Figure 1*), a disease for which now several therapeutic drugs are available.^1^ These findings exemplify how under the umbrella of HFpEF distinct and potentially treatable diseases may hide, which can be identified by appropriate cardiac imaging, to the symptomatic and prognostic benefit of the patients (*Figure 2*). In the following, we will review important diseases phenotypically manifesting as HFpEF accessible to contemporary imaging techniques, before proposing a practical diagnostic algorithm for HFpEF patients.

In a large proportion of HFpEF patients no specific underlying etiology is found except for cardiovascular risk factors like hypertension, obesity, diabetes mellitus, presence of atrial fibrillation, and others. Many of these patients would have been formerly classified as having hypertensive heart disease. This scenario calls therapeutically for the modification of such risk factors, and life style interventions like regular exercise, which have shown benefit in HFpEF.^2^ Medical therapy of “classical” HFpEF patients without a more specific etiology has shown overall modest benefits. Mineralocorticoid antagonists drugs targetting myocardial fibrosis, which had proved beneficial in heart failure with reduced ejection fraction (HFrEF), had mixed results in HFpEF. The TOPCAT trial^3^ of spironolacton showed no clinical benefit, but later analysis by geographic provenance of patients showed considerable regional variability in patient characteristics and that in patients from the Americas there was a positive effect on heart failure hospitalization and cardiovascular mortality.^4^ The guanylate cyclase stimulator vericiguat did not fulfill expectations of clinical improvement in HFpEF in a newly published large trial.^5^ However, some drugs primarily given for HFrEF, like angiotensin receptor-blockers, mineralocorticoid antagonists, and angiotensin receptor-neprilysin inhibitors may be considered in heart failure patients with ejection fraction near the lower limit or at the border zone of normal ejection fraction.

Sodium-glucose cotransporter 2 (SGLT2) inhibitors like empagliflozine and dapagliflozine improved symptoms and modestly reduced heart failure hospitalizations, but not death, in HFpEF trials.^6,7^ For more details regarding therapy of HFpEF in general the reader is referred to pertinent reviews and guidelines.^8,9^

Besides patients with “classical HFpEF” associated with cardiovascular risk factors, atrial fibrillation, there are several “mimics” or “phenocopies” of HFpEF which are amenable to specific therapies and therefore need to be identified, with imaging playing a central diagnostic role in the differential diagnosis.^10,11^ In the following, we discuss the imaging aspects of the most important specific etiologies leading to the clinical picture of HFpEF. Note that some of these diseases are sometimes classified as “restrictive cardiomyopathies” (e.g. amyloidosis, Anderson-Fabry, and hemochromatosis, as well as some hereditary cardiomyopathies), a designation which we have avoided in this review.

### Coronary artery disease including microvascular dysfunction

**Figure 3:**
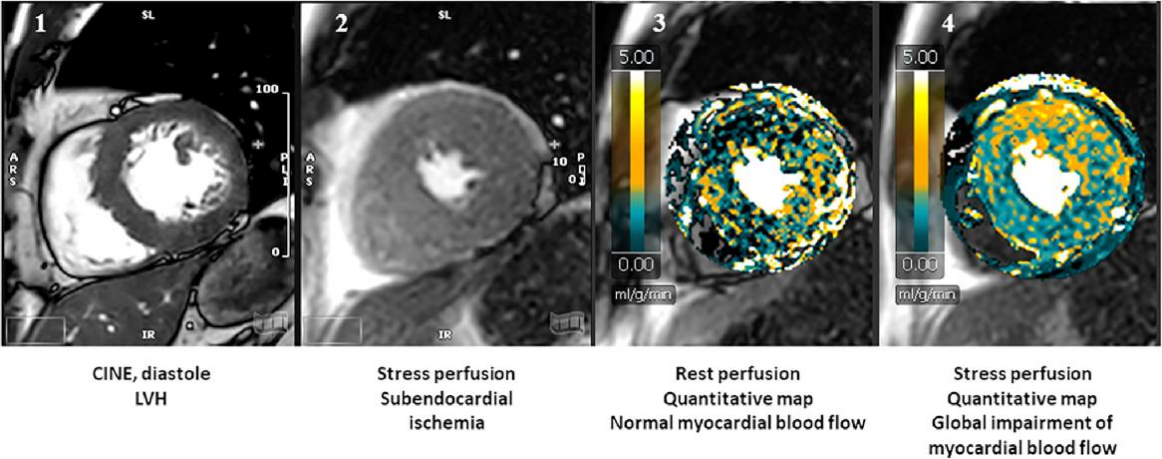
Example of HFpEF due to coronary microvascular dysfunction. Patient with heart failure symptoms and newly diagnosed left ventricular hypertrophy on echocardiography and CMR (panel 1). Panel 2 shows a thin circumferential subendocardial perfusion defect, suggestive for microvascular dysfunction. Microvascular dysfunction is clearly demonstrated by quantitative perfusion CMR (1.5 T): panel 3 shows normal perfusion at rest (green, rest myocardial blood flow 1.0 mL/g/min), while stress perfusion shows generalized impaired maximal perfusion (green-yellow; maximal myocardial blood flow 1.5 mL/g/min), resulting in a myocardial perfusion reserve of 1.5 (normal > 2.4).

Epicardial coronary artery disease (CAD) leading to loss of myocardium after infarction is a major contributor to heart failure with reduced ejection fraction. In HFpEF, however, the role of epicardial CAD is less clear. It is generally accepted though that microvascular coronary dysfunction may contribute to HFpEF and diastolic dysfunction. For example, in a study of patients with reduced coronary flow reserve by positron emission tomography (PET) and no flow-limiting epicardial coronary stenosis, diastolic left ventricular function was impaired and the risk of hospitalization for HFpEF substantially increased.^12^ Microvascular coronary dysfunction cannot be easily visualized by invasive or non-invasive coronary angiography, although postprocessing of angiography images may allow identification of increased microvascular resistance.^13,14^ Instead, a reduction of coronary flow reserve under 2-2.5, in the absence of epicardial flow-limiting stenoses, is generally accepted as sign of microvascular dysfunction, which can be obtained by PET, stress cardiovascular magnetic resonance (see *Figure 3*; ^15^), or sophisticated invasive measurements. The underlying pathomechanism of microvascular dysfunction appears to include endothelial dysfunction and inflammation, which are to some extent treatable by modification of classic cardiovascular risk factors, angiotensin-converting enzyme inhibitors, and statins. For a comprehensive discussion of the treatment of coronary microvascular dysfunction see.^16^

### Cardiac amyloidosis

**Figure 4:**
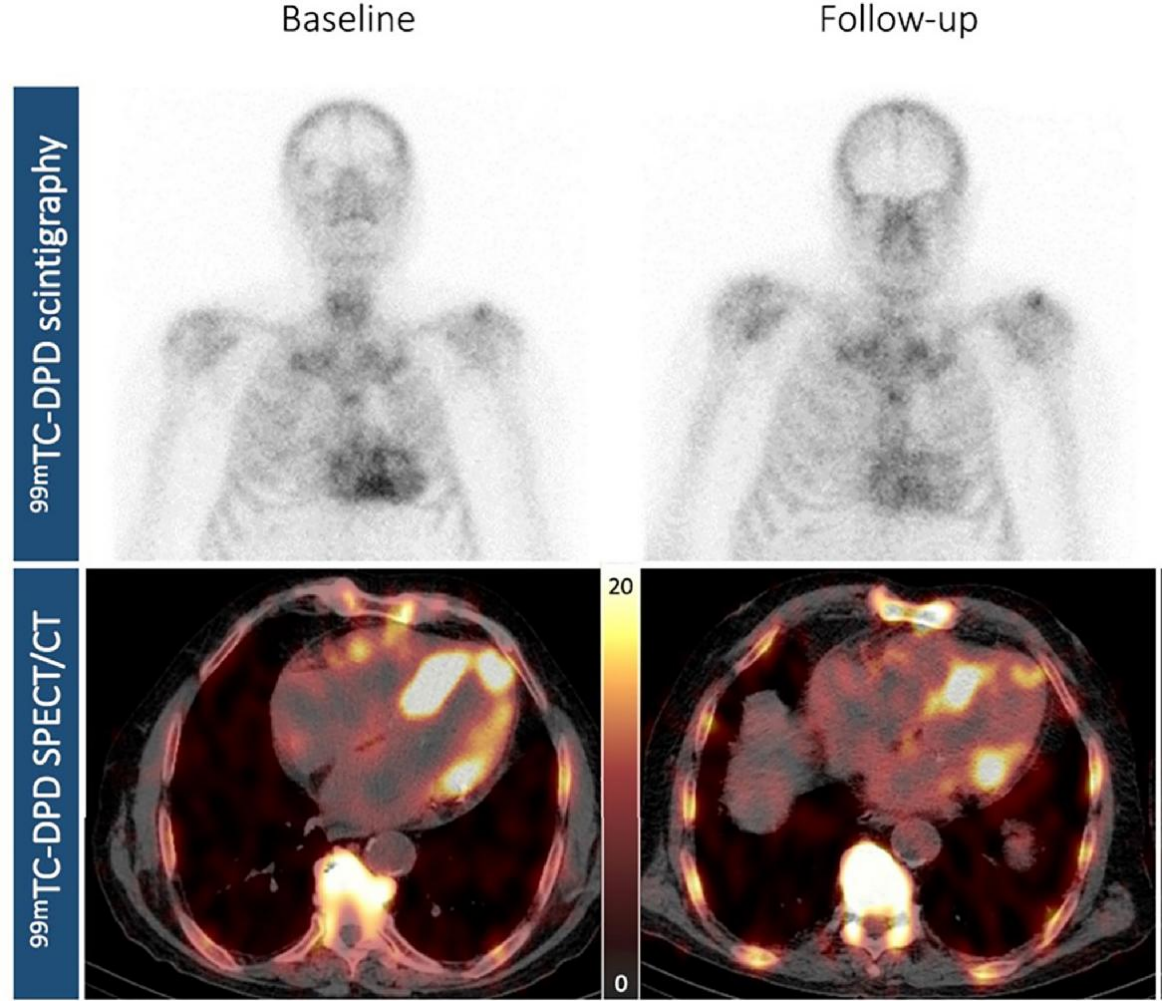
Regression of wild-type ATTR amyloidosis under tafamidis therapy. Visualization of response to tafamidis treatment (61 mg once daily) by nuclear imaging. Planar whole-body DPD-scintigraphy in the upper panels and axial ^99m^Tc-DPD-SPECT in the lower panels, with baseline findings on the left (upper left panel, Perugini grade 3, lower left panel, clear tracer uptake in the left ventricular walls; peak cardiac standardized uptake value: 12.40 g/mL) and follow-up findings after approximately 9 months on the right (upper right panel, Perugini grade 2, lower right panel, decreased myocardial uptake, peak cardiac standardized uptake value: 8.56 g/mL). Reproduced from.^22^

This disease is substantially underdiagnosed, and increasingly amenable to treatment, as well as, all reasons underlining the importance of diagnostic efforts. The typical candidate for diagnosis is a patient with echocardiographic left ventricular (LV) hypertrophy and clinical “red flags” alerting for the possibility of systemic amyloidosis, such as renal disease, carpal tunnel syndrome (especially bilateral), rupture of the long head of the biceps tendon, polyneuropathy, and, signaling cardiac involvement, low-voltage electrocardiogram (ECG), often in contrast to striking LV hypertrophy on echocardiography, atrioventricular block, atrial fibrillation, and others.^17^ The latest heart failure guidelines of US-American cardiovascular societies recommend “LV wall thickness ≥14 mm in conjunction with fatigue, dyspnea, or edema, especially in the context of discordance between wall thickness on echocardiogram and QRS voltage on ECG, and in the context of aortic stenosis, HFpEF, carpal tunnel syndrome, spinal stenosis, and autonomic or sensory polyneuropathy” as red flags.^9^ The full echocardiographic picture which is seen in late stages of the disease includes global left and often right ventricular hypertrophy, diastolic dysfunction (restrictive LV filling pattern with high E/A ratio and rapid E wave deceleration time, as well as low tissue velocities at the mitral annulus), thickening of valve cusps and interatrial septum, pericardial effusion, enlarged atria, and, on strain imaging, “apical sparing”, where apical segments continue to have near normal longitudinal function while mid and basal segments show drastically reduced strain. Global strain is also reduced. EF is often preserved due to small ventricular volumes and systolic cavity obliteration, and stroke volume is reduced. An early diagnosis of amyloidosis however is not possible by echocardiography except for the rather unspecific finding of mild or moderate left ventricular hypertrophy. More specific diagnostic imaging includes scintigraphy with bone-avid technetium (^99m^Tc) markers like DPD (“bone scan”)^18^ or PET with specific markers originally developed for Alzheimer’s disease.^19^ DPD scintigraphy, either planar or with SPECT technique, and scored visually from 0-3 grades of myocardial uptake, detects ATTR, but is unreliable for AL amyloidosis.^20^ AL amyloidosis can be identified from serum and urine protein determination. PET markers identify both ATTR and AL amyloidosis and are either scored visually or by determining quantitative parameters such as the tracer retention index or the myocardium-blood standardized uptake ratio value.^21^ PET and DPD scintigraphy in SPECT technique target directly amyloid deposition in the tissue and thus allow a degree of quantitation of cardiac amyloid, suggesting the possibility to monitor therapy by imaging, with confirmatory data emerging^22^ (*Figure 4*). Likewise, an improvement of echocardiographic global longitudinal strain over time has been reported as a response to tafamidis treatment.^23,24^ For more details see recent recommendations on amyloidosis diagnosis and management.^25,26^

Cardiovascular magnetic resonance (CMR) plays an increasing role in the diagnosis of cardiac amyloidosis (both ATTR and AL). Besides confirming or refining the morphologic echocardiographic features (e.g. hypertrophy) suggestive for amyloidosis, CMR provides myocardial tissue characterization. This is based on

- late gadolinium enhancement (LGE) imaging. Global subendocardial, or transmural, or focal/patchy LGE is typical, though not specific, for cardiac amyloidosis.
- a reverse nulling pattern on LGE imaging with myocardium getting dark before or coincident with the blood pool in Look-Locker sequences. This is due to abnormally long T1 relaxation and thus pulse inversion times in the myocardium due to extracellular expansion and amyloid deposits, blunting imaging contrast between myocardium and cavity.
- T1 mapping and mapping of extracellular volume (ECV) by pre- and post-contrast T1 measurement allow a quantitative assessment of amyloid infiltration, showing prolonged T1 relaxation times and increased extracellular volume.

Serial LGE, T1, and extracellular volume measurements have shown ability to track disease progression and response to treatment of AL or ATTR amyloidosis with different new agents; see^27^ for more details.

Retrospectively, regression of cardiac AL amyloidosis under chemotherapy has been documented on CMR by reduction in T1 and LGE as well as LV mass in a small cohort.^28^ Further, serial measurements of ECV in the heart, liver, and spleen of AL patients under chemotherapy have shown parallel regression of ECV by about 30% at 12 months in those who showed good response to hematologic therapy (i.e. substantial decrease in free light chain levels), with concomitant reduction in NTproBNP and better prognosis.^29^

It should be kept in mind that the diagnostic accuracy of the imaging modalities for the wide-ranging subgroups of cardiac amyloidosis has not yet been determined and possibly never will be, given that for some familial amyloidoses only few patients have been identified. Nevertheless, as referred to in the introduction, in unselected elderly HFpEF patients with some degree of myocardial hypertrophy a prevalence of cardiac amyloidosis of 13-14% can be expected.^1,30^

### Anderson-Fabry disease

This hereditary, X-chromosome linked deficiency of a-galactosidase A manifests in the heart as a form of hypertrophic cardiomyopathy, besides leading to kidney failure, polyneuropathy and other systemic affections. On ECG, short PQ intervals and repolarization changes (negative T waves) may be already seen during childhood. Diagnosis by blood tests is usually straightforward by assessing a-galactosidase A activity in plasma or leukocytes and genetic testing, but the disease is notoriously underdiagnosed. Due to intracellular accumulation of lysosomal sphingolipids and remodelling of the left ventricle, concentric hypertrophy of the left ventricle occurs, followed by progressive replacement fibrosis in later stages, typically beginning in the basal inferolateral wall^31,32^ and progressing from midwall to transmural distribution. Reduction in longitudinal strain (and strain rate), especially in the basal inferolateral wall, is an early sign, and over time the inferolateral wall may display wall motion abnormalities and even thinning. Enlarged, “prominent” papillary muscles are often seen. Diastolic dysfunction, in line with hypertrophy and fibrosis, is frequent. Anderson-Fabry disease has a unique signature on CMR with initially abnormally short T1 relaxation times (due to intracellular storage of sphingolipids), which later progressively increase due to extracellular fibrosis.^33,34^ This leads to “pseudonormalization” and finally prolonged T1 values especially where replacement fibrosis develops, preferentially in the basal inferolateral wall, which is detectable as presence of patchy LGE. T2 prolongation, indicating inflammatory tissue edema, also occurs in regions of replacement fibrosis. Detection of replacement fibrosis is important because reduction in hypertrophy and functional improvement under therapy seem to be less likely in the presence of localized late gadolinium enhancement.^35^ Enzyme replacement therapy with alpha-galactosidase A by intravenous infusions leads to reduction of hypertrophy, improvement in symptoms and better exercise capacity if given at an early stage.^36^ The reduction in LV mass under enzyme replacement therapy may be detected by echocardiography or CMR, but depends on whether baseline hypertrophy has been present. A recent meta-analysis of CMR findings under enzyme replacement therapy found reduction in LV mass, insignificant changes in T1 values, but a progression in late gadolinium enhancement, suggesting that once localized replacement fibrosis is present, it is not reversible.^33^ Similarly, so-called chaperone therapy with substances which modulate protein folding of the enzyme reduced hypertrophy in patients with amenable mutations (see *Figure 4*).^37,38^ Another small study found a borderline significant increase in septal T1 after 18 months of treatment with the chaperone migalastat.^39^ In a study with migalastat patients with baseline hypertrophy reduced their left ventricular mass index by 10 g/m^2^ over 30 months of treatment.^37^ Both enzyme replacement therapy and chaperone therapy seem to lack efficacy in late stages with established myocardial fibrosis, emphasizing the role of imaging to select patients likely to benefit from treatment.

### Hypertrophic cardiomyopathy

This genetically mediated cardiomyopathy is characterized by hypertrophy and disarray of cardiomyocytes, small vessel disease, myocardial fibrosis, and many morphologic abnormalities including possible outflow tract or intraventricular obstruction, apical aneurysm, mitral valve disease, and others. The echocardiographic hallmark is “unexplained” wall thickness ≥15 mm in any myocardial segment.^40^ Ejection fraction is usually high normal, although in late stages a decrease in ejection fraction occurs in a small percentage.^41^ The diagnosis of hypertrophic cardiomyopathy (HCM) of course hinges on the word “unexplained” (hypertrophy), and numerous differential diagnoses need to be considered, including hypertension, amyloidosis, Anderson-Fabry disease, athlete’s heart, and rare storage diseases. In this context, CMR imaging is very useful to confirm or improve morphologic diagnosis (including wall thickness measurements, but also other abnormalities, e.g. apical aneurysm) and to evaluate presence and extent of late gadolinium enhancement (LGE); “extensive” LGE (≥ 15% of myocardium) is regarded as a risk factor for sudden death.^42,43^ Therapeutic options were in the past limited to

- “septal reduction therapy”, i.e. alcohol ablation of a part of the basal septum, or surgical removal of septal tissue (septal myectomy), to alleviate symptoms like angina and exertional dyspnea and remove left ventricular obstruction;
- intracardiac cardioverter/defibrillator (ICD) implantation to safeguard against sudden death.

Indications for septal reduction therapy depend mainly on the presence of obstruction (peak gradient > 50 mmHg) at rest or with exercise stress and symptomatic status under maximal beta-blocker and/or calcium-antagonist therapy. Contrast echocardiography plays an important role in delineating the perfusion territory of coronary septal branches targeted for alcohol ablation to prevent damage to non-septal areas, e.g. right ventricular structures such as the papillary muscles.^44^ To assess the risk of sudden cardiac death several algorithms have been proposed which process clinical, echocardiographic, and CMR data. For details the reader is referred to current HCM guidelines and recommendations.^40,45,46^

In a major therapeutic advance, recently several new drugs have been introduced for the treatment of HCM. Mavacamten, an allosteric myosin inhibitor, has been evaluated in randomized trials of obstructive and non-obstructive HCM,^47,48,49^ with reduction in obstructive gradient, improvement in echocardiographic signs of diastolic dysfunction, less symptoms and more exercise capacity in obstructive HCM and, in non-obstructive HCM, reduction of troponin and natriuretic peptides noted. The main inclusion criterion for these studies were unexplained left ventricular hypertrophy ≥15 mm or ≥13 mm if familial HCM and, for the former study, an intraventricular pressure gradient of ≥ 50 mmHg at rest or after provocation. A second myosin inhibitor, aficamten, has also been shown to reduce obstruction and improve symptoms in HCM as defined above,^50^ and more drugs are being introduced for this indication.

### Constrictive pericarditis

Although generally not considered a form of HFpEF, constrictive pericarditis almost ideally fits the concept of a primarily diastolic cardiac dysfunction. This rare disease occurs mostly after open heart surgery, chest irradiation, or relapsing pericarditis, and is characterized by a restriction of filling of all chambers of the heart due to a calcified, thickened, and stiff pericardium. Of note, systolic left and right ventricular function are largely unaffected, except for a paradoxical early systolic septal motion due to preponderance of right over left ventricular filling. Clinical manifestations are mainly right-sided heart failure signs, such as relapsing pleural effusion, hepatic congestion, and peripheral edema. A host of echocardiographic signs have been described (see below), but in practice conclusive diagnosis of constrictive pericarditis is rarely possible by echocardiography alone.^51^ Invasive confirmation of diastolic pressure elevation and equalization is helpful and often necessary to confirm the diagnosis. Cardiac computed tomography (CT) is well suited to identify and map pericardial thickening and in particular calcification and can guide surgical pericardiectomy. However, according to experience at the Mayo clinic, a minority of patients have normal pericardial thickness and only about one fourth of all patients with constrictive pericarditis have calcifications.^52,53^ Echocardiographic signs which should raise the suspicion of constrictive pericarditis include:^54^

- inspiratory ventricular septal shift to the left and to the right in expiration;
- exaggerated respiratory variation of transvalvular flow, best seen in transmitral and transtricuspid flow during an unforced respiratory cycle;
- “annulus paradoxus”, meaning lower tissue velocities at the annular level of the lateral left ventricular and the free right ventricular wall than at the basal ventricular septum, where an e’ > 8 cm/s is typical.^54,55^ which would be unlikely in “ordinary” HFpEF with diastolic dysfunction;
- prominent reversal of expiratory late diastolic flow in the hepatic veins.

Both CT and CMR are able to demonstrate thickened pericardium, but CT allows a better appreciation of the distribution of calcification for preoperative planning of pericardiectomy.

### Iron overload cardiomyopathy (haemochromatosis)

Cardiac haemochromatosis, or iron overload cardiomyopathy, can be hereditary or, more often, secondary to frequent blood transfusions, e.g. in thalassemia patients. Cardiac manifestations include an earlier “restrictive” phenotype characterized by left ventricular diastolic dysfunction and left atrial dilatation, and a later manifestation resembling dilated cardiomyopathy.^56^ The key imaging technique in haemochromatosis is the determination of the T2* relaxation by CMR; “iron destroys magnetic resonance signal”,^57^ and therefore myocardial T2* has an inverse and histologically validated graded correlation with intracellular iron (both in the heart and the liver), with a cutoff of < 20 ms in the ventricular septum shown to be clinically and prognostically meaningful, while values < 10 ms have a very high correlation with heart failure.^58^ The more widely used CMR relaxation parameter T1 (as well as T2) is also shortened in the myocardium;^59^ for more technical detail see.^57^ The close relation of T2* values to myocardial iron load also allows to monitor iron chelation therapy by CMR, where approximately yearly exams have been found to be useful.^60^

### Valvular heart disease

Valvular disease is of course a major contributor to heart failure, and for example significant aortic stenosis with preserved ejection fraction could be categorized as a form of HFpEF. A comprehensive review of valvular heart disease^61^ however surpasses the scope of this article. Nevertheless a particular form of mitral disease, atrial functional mitral regurgitation, deserves a brief discussion, since it overlaps significantly with HFpEF and has received limited attention in the past. Atrial functional mitral regurgitation is defined by a structurally intact mitral valve, a significantly enlarged left atrium (mostly in atrial fibrillation) and left atrial annulus, and a normal sized left ventricle with preserved ejection fraction. The driving mechanism in this form of mitral regurgitation is the enlargement of the left atrial annulus, as opposed to the situation in ventricular functional mitral regurgitation, where tethering of the mitral valve by the remodelled and dilated left ventricle leads to mitral valve tenting and is the primary cause of mitral regurgitation; instead, in the atrial form of functional mitral regurgitation, there is no or minimal mitral valve tenting.^62^ Due to the absence of an increased total left ventricular stroke volume in atrial functional mitral regurgitation the severity of regurgitation is mostly mild or moderate, however the stiff atrium, especially if combined with some degree of diastolic dysfunction of the left ventricle, leads to high left atrial pressure and thus creates or enhances the clinical picture of HFpEF. Echocardiography allows to diagnose this condition; grading of severity of regurgitation is difficult, though, since some signs of increased diastolic pressure can also be interpreted as signs of mitral regurgitation severity (e.g. pulmonary arterial pressure, pulmonary venous flow, E/A ratio, etc.) and left ventricular size is by definition within normal range. CMR may be helpful to quantitate mitral regurgitation and to identify pathology of the left ventricular myocardium (e.g. by late gadolinium enhancement or abnormal T1 values). Restoration of sinus rhythm by ablation seems to be the most effective therapy for atrial functional mitral regurgitation.^63^

### Cardiac sarcoidosis

Sarcoidosis, a systemic granulomatous disease of unclear etiology, manifests typically by pulmonary granulomas. Hence, chest X-ray or CT is often the first imaging modality to show abnormalities in sarcoidosis. Cardiac involvement leads to arrhythmias, including ventricular arrhythmias and sudden death. Right bundle-branch block and advanced atrioventricular block (especially in patients under 60 years) are frequent. On echocardiography, regional wall motion abnormalities in “non-coronary” distributions may be seen, as well as localized wall thinning and aneurysms; LV systolic and diastolic and right ventricular function may be impaired, but the disease may also manifest as HFpEF. Echocardiography has well-documented low sensitivity for the diagnosis, and sarcoidosis should be considered in otherwise unexplained new heart failure, new ventricular arrhythmia/sudden cardiac death, and new advanced atrioventricular block. CMR findings include regional wall motion abnormalities and focal edema corresponding with often spotty LGE of variable location, which is an independent predictor of adverse events.^64^^18^F-fluorodeoxyglucose (FDG) PET is an excellent tool to image metabolically active granulomas, both in the heart and elsewhere in the body.^65^ Dynamic PET with perfusion tracers, such as ^15^O-water, showing corresponding perfusion defects in areas with high FDG uptake, provides complementary information on disease activity.^66^ Because of the focal nature of sarcoidosis, endomyocardial biopsy is often negative. Treatment is primarily immunosuppressive with corticoids, methotrexate or azathioprine. Therapeutic efficacy can be monitored by FDG-PET, typically after 3 months of immunosuppressive therapy, to decide whether therapy can be tapered (in the case of reduced or abolished FDG uptake) or should be escalated (in the case of persisting cardiac FDG uptake).^67,68^

### Pulmonary hypertension, right ventricular failure, and tricuspid regurgitation

Elevation of blood pressure in the lung circulation can be caused by retrograde transmission of increased diastolic LV pressures, for example in the presence of diastolic LV dysfunction, or by increased left atrial pressures in mitral valve disease (“post-capillary” pulmonary hypertension). This is even a diagnostic feature for both conditions. On the other hand, increased pulmonary arterial, and therewith right ventricular systolic, pressures may be caused by an increase in pulmonary vascular resistance due to a variety of diseases of the pulmonary circulation that ranges from pulmonary embolism to hereditary forms of pulmonary hypertension (“pre-capillary” pulmonary hypertension). All of these conditions may coexist with preserved LV ejection fraction and therefore may be diagnosed—in the presence of heart failure symptoms and signs—as HFpEF.

The diagnosis of elevated right ventricular pressures by echocardiography is usually straightforward by using maximal tricuspid regurgitation velocity to calculate the maximal transtricuspid systolic pressure gradient and adding an assumed right atrial pressure (from visual inspection of the inferior vena cava) to arrive at an estimate of right ventricular systolic pressure. Systolic ventricular septal curvature and the systolic acceleration time of the right ventricular outflow tract flow signal can also be used to estimate right ventricular systolic pressure. Note however that these parameters are not able to distinguish pre- and post-capillary pulmonary hypertension. Early or mid-systolic notching of the forward flow signal in the right ventricular outflow tract suggests pulmonary obstruction. It is however impossible to calculate pulmonary vascular resistance with confidence from echocardiography. Further, in the presence of very high right atrial v-waves due to severe tricuspid regurgitation, the calculation of right ventricular systolic pressure in the described way becomes unreliable since right atrial peak v-wave pressure may far exceed 15 mmHg. Other imaging modalities like CT or CMR only provide qualitative signs of pulmonary hypertension such as dilatation of the right ventricle or main pulmonary artery. Hence, diagnosis and therapy monitoring of primary pulmonary hypertension necessitates invasive measurement of right-sided pressures and pulmonary blood flow.

Primary pulmonary arterial hypertension, or group 1 PAH, is treatable by several classes of drugs including calcium channel blockers, endothelin antagonists, phosphodiesterase inhibitors and guanylate cyclase stimulators; up-titration of therapy requires invasive evaluation of pulmonary pressures and vascular resistance. For more details see pertinent guidelines.^69^

A specific form of pulmonary hypertension, chronic thromboembolic pulmonary hypertension (now called group 4 PAH), which is amenable to catheter intervention or surgery, can be identified by perfusion/ventilation scintigraphy, CMR perfusion study, or CT of the pulmonary vasculature. The latter enables the visualization of pulmonary arterial stenoses or obstruction, e.g. by chronic pulmonary embolism. For the complete evaluation of chronic thromboembolic pulmonary hypertension, invasive pulmonary angiography continues to be necessary, especially for the planning of interventions.

Finally, severe tricuspid regurgitation not caused by left heart disease leads to systemic venous congestion and may be mistaken as HFpEF. Diagnosis is straightforward by echocardiography and work-up as well as therapeutic options are discussed in current guidelines for management of valvular diseases.^61^

### How should we image hFpEF patients “wisely”?

The categorization of newly diagnosed heart failure patients into the different EF-related groups represents the first pivotal step in heart failure management.^8^ This initial stratification is almost always done by echocardiography, which in one complete examination provides—beyond EF—further crucial information:

- LV regional function and LV global strain
- diastolic LV function
- presence of LV hypertrophy
- right ventricular size and function, and pulmonary systolic pressure
- left atrial size and function
- valvular heart disease
- pericardial disease (e.g. constrictive pericarditis)

Bearing in mind that the phenotype of HFpEF includes a “classic” form believed to be caused or triggered by cardiovascular risk factors, but also, as reviewed in this article, a considerable number of different specific diseases (“phenocopies”) requiring specific therapies, such specific diagnoses should always be considered. Abnormalities found in the baseline echocardiographic examination should trigger potential further exploration by other imaging modalities (e.g. nuclear imaging or CMR in response to “unexplained” LV hypertrophy/thickening or to “red flags” for amyloidosis), laboratory tests (such as immune fixation electrophoresis for AL amyloidosis, or genetic characterization for hypertrophic cardiomyopathy), and in rare cases invasive investigations such as right heart catheterization (e.g. to evaluate the pulmonary circulation in suspected or treated pulmonary arterial hypertension) or endomyocardial biopsy.^70^ For a number of therapies, serial imaging can add objective evidence of functional or structural improvement, as discussed in the sections above. It should be kept in mind, however, that sometimes unchanged imaging data (e.g. degree of hypertrophy) over time may represent a halting of progression and not therapeutic failure.

## Conclusion

- Multimodality imaging can, in a substantial number of patients, identify or rule out specific etiologies (“phenocopies”) of HFpEF and thus open specific therapeutic options.
- The choice of imaging depends on the individual clinical pre-test likelihood, e.g. “red flags”, as well as availability and experience.
- In most diseases, serial cardiac imaging allows to at least to partially assess the objective efficacy of treatment, with consequences for escalation, change, or withdrawal of therapy.
